# Head Rules Over the Heart: Cardiac Manifestations of Cerebral Disorders

**DOI:** 10.5005/jp-journals-10071-23208

**Published:** 2019-07

**Authors:** Ajay Prasad Hrishi, Karen Ruby Lionel, Unnikrishnan Prathapadas

**Affiliations:** 1,3 Division of Neuroanesthesia, Department of Anesthesiology, Sree Chitra Tirunal Institute for Medical Sciences and Technology, Thiruvananthapuram, Kerala, India; 2 Department of Anesthesiology, Christian Medical College, Vellore, Tamil Nadu, India

**Keywords:** Neurocardiac axis, Neurocardiology, Neurological disorders

## Abstract

The Brain-Heart interaction is becoming increasingly important as the underlying pathophysiological mechanisms become better understood. “Neurocardiology” is a new field which explores the pathophysiological interplay of the brain and cardiovascular systems. Brain-heart cross-talk presents as a result of direct stimulation of some areas of the brain, leading to a sympathetic or parasympathetic response or it may present as a result of a neuroendocrine response attributing to a clinical picture of a sympathetic storm. It manifests as cardiac rhythm disturbances, hemodynamic perturbations and in the worst scenarios as cardiac failure and death. Brain-Heart interaction (BHI) is most commonly encountered in traumatic brain injury and subarachnoid hemorrhage presenting as dramatic electrocardiographic changes, neurogenic stunned myocardium or even as ventricular fibrillation. A well-known example of BHI is the panic disorders and emotional stress resulting in Tako-tsubo syndrome giving rise to supraventricular and ventricular tachycardias and transient left ventricular dysfunction.

In this review article, we will discuss cardiovascular changes caused due to the disorders of specific brain regions such as the insular cortex, brainstem, prefrontal cortex, hippocampus and the hypothalamus; neuro-cardiac reflexes namely the Cushing's reflex, the Trigemino-cardiac reflex and the Vagal reflex; and other pathological states such as neurogenic stunned myocardium /Takotsubo cardiomyopathy. There is a growing interest among intensivists and anesthesiologists in brain heart interactions as there are an increasing number of cases being reported and there is a need to address unanswered questions, such as the incidence of these interactions, the multifactorial pathogenesis, individual susceptibility, the role of medications, and optimal management.

**Key Messages:**

BHI contribute in a significant way to the morbidity and mortality of neurological conditions such as traumatic brain injury, subarachnoid hemorrhage, cerebral infarction and status epilepticus. Constant vigilance and a high index of suspicion have to be exercised by clinicians to avoid misdiagnosis or delayed recognition. The entire clinical team involved in patient care should be aware of brain heart interaction to recognize these potentially life-threatening scenarios.

**How to cite this article:**

Hrishi AP, Lionel KR, Prathapadas U. Head Rules Over the Heart: Cardiac Manifestations of Cerebral Disorders. Indian J Crit Care Med 2019;23(7):329–335.

## INTRODUCTION

The interaction between the brain and the heart is becoming increasingly important as the underlying mutual mechanisms become better understood. In 1985, Natelson described a new interdisciplinary area termed “Neurocardiology”, which examined the interaction between the brain, autonomic nervous system and the cardiovascular system in pathological states.^1^ “Neurocardiology” is a new field which explores the pathophysiological interplay of the brain and cardiovascular systems.^1,2^ Brain-heart cross-talk presents due to the direct stimulation of certain areas of the brain, leading to a sympathetic or parasympathetic response or it may present as a result of a neuroendocrine response attributing to a clinical picture of a ‘sympathetic storm’. It manifests as cardiac rhythm disturbances, hemodynamic perturbations and in the worst scenarios as cardiac failure and death. Brain-heart interaction (BHI) is most commonly encountered in traumatic brain injury (TBI) and subarachnoid hemorrhage (SAH) presenting as dramatic electrocardiographic changes, such as QT prolongation and ventricular tachyarrythmias and in worse scenarios as neurogenic stunned myocardium.^3,4^ Another example of BHI is panic disorders and emotional stress leading to Tako-tsubo syndrome giving rise to supraventricular and ventricular tachycardias with ensuing transient left ventricular dysfunction.^5,6^ In this review article on BHI, we will discuss cardiovascular changes caused due to the stimulation of specific brain regions, neuro-cardiac reflexes and neurogenic stunned myocardium /Tako-tsubo cardiomyopathy.

## SPECIFIC BRAIN REGIONS AND CARDIOVASCULAR CHANGES

### The Neuro-cardiac Axis

Neuroimaging with positron emission tomography (PET) and functional magnetic resonance imaging (fMRI), reveal a complex set of neural pathways and interactions which are termed as the ‘neuro-cardiac axis’. The ‘neuro-cardiac axis’ consists of the prefrontal cortex, amygdala, insular cortex, the anterior cingulate cortex and the brainstem all which are involved in the control of the autonomic nervous system.^7,8^

#### Cardiovascular Changes and the Insular Cortex

The insular cortex located deep at the base of the sylvian fissure plays a vital role in controlling the sympathetic and the parasympathetic tone. An example of the effect of the insula on the cardiovascular system (CVS) is typically manifested in middle cerebral artery stroke victims. These patients are at an elevated risk of sudden cardiovascular death and autonomic alterations.^9^

**Fig. 1:**
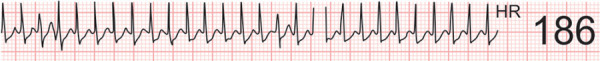
ECG (electrocardiogram) showing supraventricular tachyarrythmia in a patient who has had left insular stroke

In the intraoperative setting, surgical stimulation of the rostral posterior insula culminates in pure tachycardia, whereas the stimulation of the caudal posterior insula results in bradyarrhythmias. There is also lateralization of cardiac control by the insula i.e.; the right insular regions predominantly regulate the sympathetic tone, and the left insula regulates parasympathetic cardiac manifestations.^10,11^ Intraoperatively, surgical manipulation of left insular cortex results in bradycardia or hypotension, whereas stimulation of the right insula causes tachycardia with a pressor effect.^11^ In the setting of cerebrovascular accidents (CVA), patients with left insular stroke commonly experience a sympathetic predominance resulting in cardiac dysrhythmias and cardiac wall motion abnormalities (Fig. 1). Also, a CVA involving the right insula can result in neurogenic stunned myocardium (NSM) and more complex arrhythmias than CVA involving other localizations.

#### Cardiovascular Changes and Brainstem

The brain stem structures, especially the rostroventrolateral medulla (RVLM) and the nucleus of the solitary tract (NST) in the medulla, plays a vital role in the regulation of cardiac function.^12,13^ The NST receives afferents from baroreceptors and the cranial nerves, including the vagus which communicates the visceral sensorial information. The RVLM is mainly constituted by excitatory neurons, which are responsible for the initiation of the sympathetic response. Furthermore, the RVLM, along with the external lateral parabrachial nucleus of the pons, is involved in the CNS processing of excitatory cardiovascular reflexes resulting in cardiac sympathetic stimulation.^14,15^ The brainstem is an essential regulator of cardiovascular responses to the interactive or exteroceptive environment, as well as vagal and sympathetic nerve activity.^16^ Patients having brainstem lesions can present with autonomic dysfunction, ventricular arrhythmias, T wave inversion, bradyarrhythmias, myocardial infarction (MI), and sudden cardiac death.^17,18^

A close association between various forms of cardiac arrhythmias and brainstem lesions caused due to transtentorial herniation have been identified. In a setting of brainstem ischemia or raised ICP; respiratory arrest, bradycardia and a rise in blood pressure are registered the moment ischemia reached the lower pons. Further advance of the ischemic front into the lower medulla oblongata lead to an abrupt change from bradycardia to tachycardia. In intraoperative settings, the stimulation of the floor of the fourth ventricle during posterior fossa surgeries can result in bradyarrhythmias and the stimulation of the periventricular zone causes tachyarrythmias and hypertension.

#### Cardiovascular Changes and Prefrontal Cortex

High-frequency heart rate variability (HF-HRV) of a subject during a resting state is said to reflect the degree of vagal blocking of the sympathetic activity. HF-HRV can be used to assess the interaction of cardiovascular changes with the nervous system following a cerebral injury after surgery.^19^ The prefrontal cortex region is the main contributor to the nervous network related to HF-HRV. This is evidenced by the impaired autonomic cardiac control that occurs following injury to the prefrontal cortex. Patients with lesions in the prefrontal cortex or ischemia of the frontal lobe can present with parasympathetic features such as bradycardia, hypotension, and HF HRV in the ECG, which is attributed to sympathetic activity blockade.^19,20^

#### Cardiovascular Changes and Hippocampus

Hippocampus is a part of the ‘neuro-cardiac axis’, and any pathology involving the hippocampus such as seizures, infarct or tumours could result in autonomic dysregulation. Hemispheric brain infarcts involving hippocampus can result in new-onset hypertension, atrial fibrillation, myocardial infarction and cardiac failure.^21^ Hippocampal infarcts have a poor outcome as evidenced by the postmortem analysis, revealing an association of hippocampal infarcts with heart failure and sudden cardiac death.^22^ Seizures attributed to hippocampal lesions are associated with sudden unexpected death owing to the severe sympathetic dysfunction resulting in acute MI and heart failure.

#### Cardiovascular Changes and the Hypothalamus

Hypothalamic stimulation in an intraoperative setting can lead to a plethora of cardiovascular disturbances.^23^ Intraoperative stimulation of the lateral hypothalamus produces hypertension and tachyarrthymias, the common presentation being atrial tachyarrhythmias. Stimulation of the anterior hypothalamus produces bradycardia, whereas stimulation of the posterior hypothalamus results in tachycardia and sympathetic overactivity. This is commonly encountered during endoscopic third ventriculostomy (ETV) procedures or surgeries involving the seller suprasellar pathologies such as craniopharyngioma or pituitary macroadenomas. The sympathetic responses could be prevented by the performing a C_2_ nerve ablation and stellate ganglion block. The parasympathetic responses can be prevented by performing blocking the vagal nerve.^23^ This is the rationale by which, stellate ganglion blockade has been explored in the management of neurogenic stunned myocardium and vasospasm in SAH settings as it reduces the intensity of the sympathetic storm.

#### Central Nervous System (CNS) Trigger Zones

**Fig. 2:**
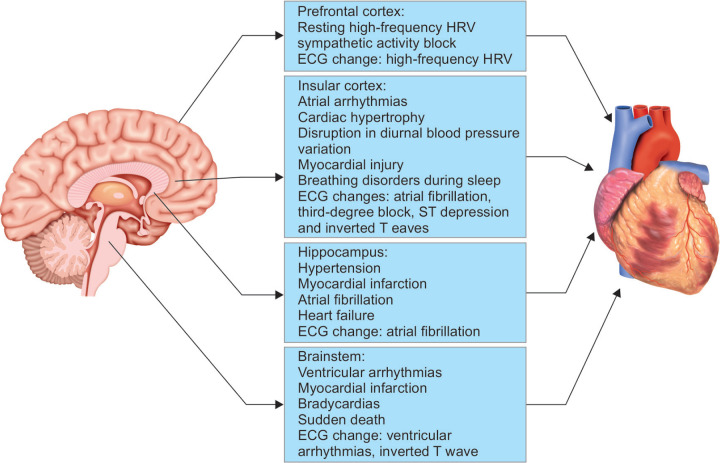
This figure shows the central and peripheral mechanism of the heart and brain interaction and the clinical symptoms and ECG changes which are encountered on triggering specific brain regions. Among the regions insular cortex, prefrontal cortex, hippocampus, and brainstem play an important role in interplay between the nervous system and the cardiovascular system

Stimulation of specific areas of the CNS stimulates the sympathetic nervous system resulting in catecholamine release. Ischemia to the hypothalamus, medulla oblongata and its surrounding tissues are responsible for the centrally mediated ‘sympathetic storm’. The structures implicated are the caudal ventrolateral medulla (A_1_ catecholaminergic neurons), the tractus solitarius, the dorsal motor vagal nucleus and the posterior hypothalamus. Stimulation of these trigger zones by blood, thrombus, inflammatory mediators or an ischemic/surgical insult can result in two pathophysiological mechanisms. Firstly, a centrally mediated profound sympathetic discharge leading to a precipitous loss of vasomotor homeostasis, intense pulmonary vasoconstriction and increased cardiac rate and contractility; secondly, an inflammatory mediator-related increase in vascular permeability.^24^ These CNS trigger zones have been incriminated in the pathogenesis of NSM, and neurogenic pulmonary oedema in various intracranial pathologies such as SAH, TBI, cerebrovascular accidents (embolic stroke, intracerebral hemorrhage), epilepsy, and in conditions causing acute rise in intra cranial pressure (e.g., blocked ventriculo peritoneal shunt).^24,25^ The summary of the central and peripheral mechanism of the brain-heart interaction, the clinical symptoms and ECG changes which are encountered while triggering specific brain regions is illustrated in Fig. 2.

### Neuro-cardiac Reflexes

#### Cushing's Reflex

A rise in intra cranial pressure (ICP) initiates the complex mechanism of the Cushing's reflex. Since CSF is present in a closed space (skull), increased ICP consequently increases the CSF pressure. The CSF pressure eventually rises to the point wherein it gradually exceeds the mean arterial pressure (MAP). When the ICP exceeds the MAP, there is diminished blood supply to the brain, resulting in cerebral ischemia.^26^ This ‘CNS ischemia’ is perceived by the hypothalamus which triggers a ‘CNS ischemic response’. The hypothalamic activation causes a sympathetic overdrive, resulting in increased cardiac output, peripheral vasoconstriction and increased arterial blood pressure. Thus, the MAP exceeds the ICP, and blood flow to the CNS is restored. This constitutes phase one of the Cushing's response. Phase two is initiated when the resultant increased arterial blood pressure stimulates the baroreceptors in the carotid bodies, resulting in a reflex reduction in the heart rate sometimes drastically to the point of bradycardia.^26^ Patients with raised ICP as in TBI, stroke and intracranial space-occupying lesions (ICSOL) maintain their blood pressure due to the Cushing's reflex. Thus, when the ICP is acutely reduced by decompressive surgical management and CSF diversion procedures (e.g. external ventricular drain) or with the institution of anesthesia and cerebral protection, there could be a sudden precipitous fall in the SBP and MAP.

#### Trigemino-cardiac Reflex or Oxygen Conserving Reflex

The trigemino-cardiac reflex (TCR) is initiated by the stimulation of the sensory branches of the trigeminal nerve. This stimulation results in parasympathetic dysrhythmia (bradycardia), hypotension, apnea and gastric hypermotility. The afferent pathway of the reflex arc is constituted by the branches of the sensory division of trigeminal nerve sending neuronal signals through the gasserian ganglion to the sensory nucleus of the trigeminal nerve. This afferent pathway communicates to the motor nucleus of the vagus through the short inter-nuncial nerve fibres in the reticular formation. The reflex stimulation of the motor nucleus of the vagus nerve results in bradycardia, hypotension and apnea. In neurosurgical setting, TCR is encountered during craniofacial surgery, balloon-compression/rhizolysis of the trigeminal ganglion and tumour resection in the cerebellopontine angle. It is also commonly encountered during ophthalmological procedures, especially in squint correction surgeries.^27^

Off late, the physiological role of this brainstem-reflex has been widely explored by researchers in a neuroprotective context. It has been evidenced that the TCR is a central neurogenic reflex which leads to rapid cerebrovascular vasodilatation, the trigger being the excitation of oxygen-sensitive neurons in the rostral ventrolateral medulla oblongata (RVLM). This physiological response, causes a redistribution of the cerebral and systemic circulation, thereby diverting the systemic blood to the brain and increase the cerebral blood flow (CBF). Hence, the endogenous neuroprotective role of TCR may extend beyond the known clinical appearance of the TCR. Furthermore, TCR which initially was considered as a pathophysiological entity, has now been rediscovered as a physiological oxygen conserving reflex.^27^ Further research in this area might help in the application of TCR in a neuroprotective role in the prevention of secondary neurological injuries in acutely injured brain.

**Figs 3A and B:**
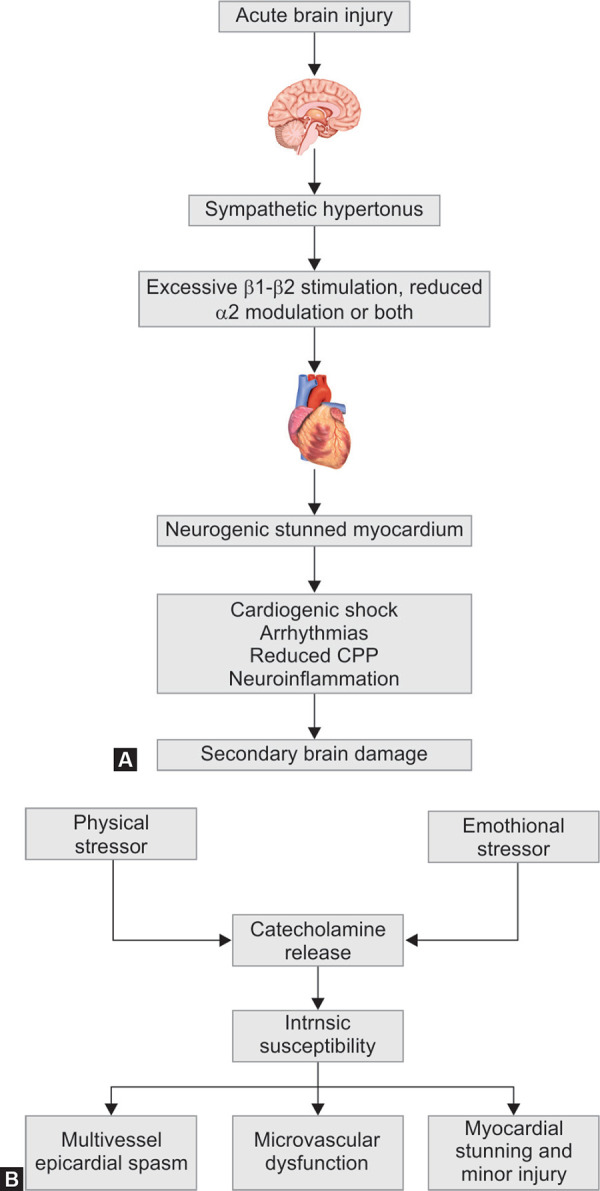
(A) Schematic illustration of the ‘catecholamine hypothesis mechanism’ responsible for neurogenic stress cardiomyopathy after acute brain injury; (B) Schematic illustration of the pathophysiology for ‘Takotsubo Cardiomyopathy’. Both these pathologies can induce secondary brain damage by impaired systemic and brain homeostasis. Abbreviations :CPP, cerebral perfusion pressure

#### Vagal Reflex

Vagal reflex constitutes sudden bradycardia, depression of cardiac function and arteriolar vasodilatation resulting in hypotension. The bradycardia component is a result of the sudden augmentation of efferent vagal activity, and the hypotension is caused by the sudden cessation of the sympathetic activity causing relaxation of arterial resistance vessels.^28^ Two different trigger pathways have been identified to the causation of this reflex: the first, being the central neurogenic pathway originating from the hypothalamus and the second, being the peripheral pathway originating from the heart. The central pathway is triggered either by an emotional stress or by a painful stimulation resulting in the hypothalamic activation of the medullary cardiovascular centre resulting in stimulation of the motor nucleus of the vagus. The peripheral pathway could be triggered either by an increased inotropic state of the heart or by reduced central blood volume or a combination of both which in turn stimulate the ventricular mechanoreceptors causing vasodilatation and bradycardia.^28^ The vagal reflex is commonly encountered in intraoperative settings during the tumour resection in the cerebellopontine angle and procedures in the head and neck region.

### Neurogenic Stunned Myocardium (NSM)/Takotsubo Cardiomyopathy (TC)

“Neurogenic stunned myocardium” (NSM), or “neurogenic stress cardiomyopathy” (NSC) is a clinical syndrome that is triggered by severe neurological insults such as TBI, SAH, CVA or refractory seizures.^29^

#### Pathophysiology of Neurologic Stunned Myocardium/Takotsubo Cardiomyopathy

Both NSM and TC are a part of the stress-related cardiomyopathy spectrum. Tako-tsubo cardiomyopathy has a typical presentation with apical and mid-ventricular dysfunction. NSM has a significant overlap with TC when it comes to clinical presentation, pathophysiology and in terms of reversibility. In NSM, the underlying pathophysiology of myocardial dysfunction is attributed to the catecholamine storm which triggered by an acute neurological injury (Fig. 3A). Whereas, Takotsubo cardiomyopathy, in contrast, is a stress-related cardiomyopathy syndrome caused due to emotional or physical stress. TC is also frequently encountered in postpartum and perioperative settings (Fig. 3B).^30^

Stimulation of specific areas of the CNS stimulates the sympathetic nervous system resulting in catecholamine release. Ischemia to the hypothalamus, medulla oblongata and its surrounding tissues are responsible for the centrally mediated sympathetic discharge. The structures implicated are the caudal ventrolateral medulla (A_1_ catecholaminergic neurons), the tractus solitarius, the dorsal motor vagal nucleus and the posterior hypothalamus. Stimulation of these areas by blood, thrombus and inflammatory mediators or an ischemic/surgical insult can result in two pathophysiological mechanisms. Firstly, a centrally mediated profound sympathetic discharge leading to a precipitous loss of vasomotor homeostasis, intense pulmonary vasoconstriction and increased cardiac rate and contractility; secondly, an inflammatory mediator-related increase in vascular permeability.^24^ These CNS trigger zones have been incriminated in the pathogenesis of NSM.^31^ In the case of Takotsubo cardiomyopathy, the hypothalamo-pituitary-adrenal axis and the sympathoadrenomedullary axis are the two biological systems that are implicated the activation of which occurs during a stress response, resulting in a catecholamine surge.

Many causation theories for stress cardiomyopathy syndromes have been described: (i) transient multivessel coronary artery spasm; (ii) microvascular dysfunction; (iii) aborted myocardial infarction with spontaneous coronary thrombus lysis; and (iv) the ‘catecholamine hypothesis’.^32^

**Fig. 4:**
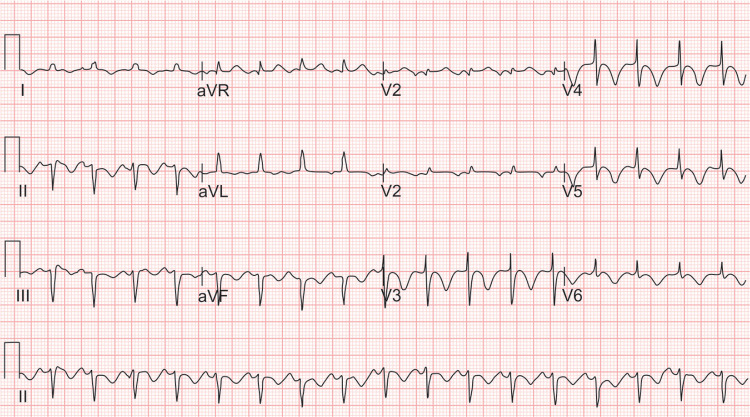
ECG (electrocardiogram) showing global ST segment depression and T inversion in all the leads which is encountered in both neurogenic stunned myocardium and takotsubo cardiomyopathy.

In patients experiencing severe sympathetic storm, a typical tissue lesion named as “myocardial contraction band necrosis” has been described. This contraction band necrosis is characterized by the hypercontraction of the sarcomeric myofibrils, interstitial mononuclear infiltration and presence of eosinophilic transverse bands. The catecholamine overload experienced by patients in NSM results in myocardial disarray due to increased extracellular matrix proteins, inflammatory cell infiltration, fibrotic changes contraction band necrosis and elevated collagen I:III ratio.^32^

#### Diagnosis of Neurogenic Stunned Myocardium

Clinical presentation of NSM mimics that of acute myocardial infarction (MI), and it is difficult to differentiate between both, especially during the first hours of admission when the patient might present with minimal neurological symptoms and signs. Initiation of anticoagulants in such presumed cases of MI could result in deleterious outcomes such as rebleed in patients having SAH or ICH. This situation is especially encountered when patients present with cardiac manifestations, such as ischemic ECG changes, arrhythmias or Adams - Stokes and are later diagnosed as having NSM.^33^ ECG changes commonly encountered in NSM are Q-T interval prolongation or long Q-T syndrome, ST segment changes, T wave inversion (Fig. 4) or in rare scenarios torsade de points, ventricular or supraventricular arrhythmias. On further evaluation, these patients can have left ventricular regional wall motion abnormalities (RWMA), elevated troponins, and B-type natriuretic peptide.^34^

### How is NSM/TC Different From Primary Cardiac Disease?

#### Pathological Presentation of NSM/TC

**Fig. 5:**
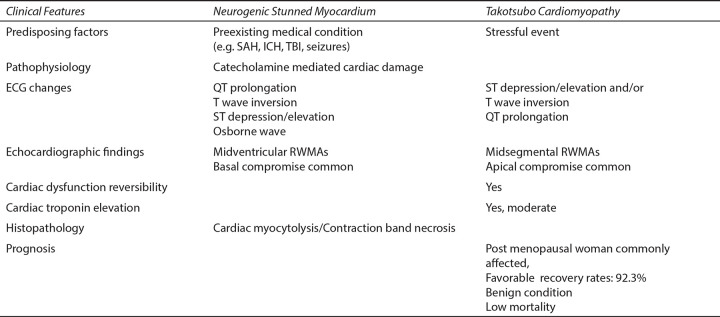
Table showing the differences between stress cardiomyopathy (Takotsubo cardiomyopathy) and neurogenic stunned myocardium. Abbreviations: ECG, electrocardiogram; ICH, intracranial hemorrhage; RWMAs, regional wall motion abnormalities; SAH, subarachnoid hemorrhage; TBI, traumatic brain injury.

There are few significant differences between the pathological presentation of MI and that of NSM and TC. In MI, the cells die in a relaxed state with the typical polymorphonuclear cell response and ischemic necrosis in the affected vascular territory. Whereas in NSM, the cells die in a hyper-contracted state and are associated with contraction bands and myofibrillar lesions, which are visible within minutes of onset along with early calcification of the lesion. In NSM, the lesions appear close to the cardiac nerves and do not follow the vascular anatomy. Furthermore, in NSM and TC, the RWMA's extend beyond the single epicardial vascular distribution, and these changes are reversible.^33^ Takotsubo cardiomyopathy presents as dyskinesia of the apical regions of the left ventricle resulting in the typical “apical ballooning” in echocardiography. This pathognomonic presentation is because the left ventricle (LV) apex is more vulnerable to catecholamine-mediated toxicity when compared to basal regions of the myocardium as there is a more significant proportion of ß_2_ adrenergic receptors with increased ß_2_ receptor- sensitivity in the apex than in basal cardiomyocytes. “Apical sparing” pattern has been noted in over 50 % of the cases of NSM. In this setting, there is RWMA's in the basal and mid-ventricular portions of the Left ventricle with sparing of the apex. The apical sparing could be due to the paucity of sympathetic nerve terminals in the LV apex.^31–33^ The significant differences between neurogenic stunned myocardium and Tako-tsubo cardiomyopathy are further highlighted in Fig. 5.

#### Management of Neurogenic Stunned Myocardium and Takotsubo Cardiomyopathy

The main concerns in the perioperative setting will be hemodynamic instability as a result of arrhythmias, cardiogenic shock and pulmonary oedema. Patients with apical ballooning are at high risk of formation of LV clots, which are at high risk of a systemic embolism, that could hamper the CBF causing further ischemic brain injury. A significant percentage of patients present with dynamic LV outflow obstruction, especially with a hyper-contractile small LV resulting in hemodynamic collapse.^32^ In most scenarios, the presentation of NSM/TC will be transient and reversible, thereby requiring only supportive management. NSM, sometimes can have severe presentation such as cardiac dyskinesias and arrhythmias which could be detrimental to the patient outcome, if there is an associated acute brain injury as the maintenance of the stable hemodynamics is essential to avoid secondary neuronal injury. In patients with SAH, NSM can result in the postponement of surgical management as the patient may not be hemodynamically stable. Also, the myocardial dysfunction and hemodynamic instability leading to hypotension and decreased oxygenation could be devastating during periods of cerebral vasospasm.^35^ It can also hamper the initiation of hypertensive therapy in patients suffering from cerebral vasospasm, thereby resulting in a poor neurologic outcome.

There is a robust physiological evidence to warrent the use of beta blockers in such conditions to counter the increased sympathetic activity and supraphysiologic levels of plasma catecholamines. Beta blockers also have a protective effect in the cerebrovascular system, which is brought about by the reduction of inflammatory mediators such as tissue necrosis factor (TNF) levels along with the membrane stabilising and antioxidant effects. Neurogenic stunned myocardium may benefit from inotropic drugs to maintain an equilibrium between myocardial oxygen demand and supply. As epinephrine-mediated stunning is the recognized causation for NSM, the use of epinephrine in such patients may worsen the negative inotropism further.^34–35^ Thus, cautious use of sympathomimetic drugs such as noradrenaline and adrenaline is advised in patients with NSM and TC as their effects are unpredictable due to the dysfunctional myocardial response to catecholamine stimulation. Levosimendan, a calcium sensitizer, is a non-catecholamine inotrope, that can be used to improve myocardial contractility in NSM and TC as it stabilises the troponin C and enhances the calcium sensitivity of cardiac myofilaments. In addition to increasing the myocardial systolic performance, it also improves coronary perfusion and has an anti-apoptotic and anti-stunning effect. Levosimendan has been reported to be successful in improving the cardiac output in patients presenting with Takotsubo cardiomyopathy.^33^

#### Prevention of Perioperative and Critical Care Takotsubo Cardiomyopathy

Though many anesthetic drugs theoretically protect against neurological stress, many cases of Tako-tsubo cardiomyopathy have been described during the perioperative period and in critically ill patients in intensive care units (ICU). The stress caused by tracheal intubation, mechanical ventilation, pain etc. and the consequent sympathetic reflex stimulation could explain the occurrence of Takotsubo cardiomyopathy in these settings. TC has also been reported in patients suffering from anaphylaxis, meperidine-induced histamine release, transfusion reactions, and infusion of adrenergic drugs, which is very commonly used in the perioperative and ICU settings. Though regional anesthesia reduces the occurrence of sympathetic stimulation arising from surgical stimulation and provides optimal postoperative pain control, TC has also been described in patients who underwent spinal anesthesia. Anesthesiologists and intensivists should, therefore, be aware of the possibility of this syndrome during the entire perioperative period and should be vigilant in the patients exposed to acute stressful events.^36–38^

Although there is no consensus regarding the management of these patients, it is crucial that any potential triggering event that could result in a catecholamine surge and consequent cardiac dysfunction be avoided during the entire perioperative/intensive care period. A brief laryngoscopy, smooth emergence, and extubation could reduce the incidence of TC. Good anxiolysis in the preoperative period, optimal depth of anesthesia/sedation before any intense stimulation, and optimal analgesia may help in reducing the incidence of NSM/TC. Opioids and the central alpha_2_-inhibitor- dexmedetomidine, both of which targets the locus coeruleus, may be the appropriate drugs in this setting. Both these group of drugs protect against psychological stress and anxiety, offers analgesia and provide hemodynamic stability, especially in intraoperative and critical care settings.^39^

NSM and TC are increasingly being recognized in the acute phase of severe brain injury and in the perioperative/ICU period, with neurogenic stunned myocardium being the best known clinical life-threatening expression of brain heart interaction. This phenomenon may also be the causation in the pathogenesis of sudden unexplained deaths in adults, sudden infant death, sudden death during asthma attacks, refractory seizures and sudden death during the alcohol withdrawal syndrome, all of which may be linked by stress and catecholamine toxicity.

Recent research is focusing on how to identify the patients at risk of developing NSM and TC, at an early stage, after acute brain injury/stress and how to protect them. Mandatory screening and monitoring of the cardiovascular function should be considered at the time of admission of patients with acute neurological insult, and careful re-evaluation should be planned accordingly. Prompt diagnosis of stress-related cardiomyopathy syndromes is vital for the prevention of catastrophic outcomes.

## CONCLUSION

To recapitulate, there is compelling evidence to suggest that the brain-heart interaction is the causation of major cardiac pathologies seen in patients with a neurological insult. These interactions may contribute in a significant way to the mortality rates of many primarily neurological conditions such as traumatic brain injury, subarachnoid hemorrhage, cerebral infarction and status epilepticus. Constant vigilance and a high index of suspicion have to be exercised by clinicians to avoid misdiagnosis or delayed recognition. The entire clinical team involved in patient care should be aware of brain heart interaction to recognise these potentially life-threatening scenarios.
